# A prediction-focused approach to personality modeling

**DOI:** 10.1038/s41598-022-16108-3

**Published:** 2022-07-25

**Authors:** Gal Lavi, Jonathan Rosenblatt, Michael Gilead

**Affiliations:** 1 Department of Industrial Engineering and Management, Ben-Gurion University of the Negev, Beersheba, Israel; 2 Pagaya Technologies, Tel Aviv, Israel; 3 School of Psychological Sciences and Sagol School of Neuroscience, Tel Aviv University, Tel Aviv, Israel

**Keywords:** Human behaviour, Social neuroscience

## Abstract

In the current study, we set out to examine the viability of a novel approach to modeling human personality. Research in psychology suggests that people’s personalities can be effectively described using five broad dimensions (the Five-Factor Model; FFM); however, the FFM potentially leaves room for improved predictive accuracy. We propose a novel approach to modeling human personality that is based on the maximization of the model’s predictive accuracy. Unlike the FFM, which performs *unsupervised* dimensionality reduction, we utilized a *supervised* machine learning technique for dimensionality reduction of questionnaire data, using numerous psychologically meaningful outcomes as data labels (e.g., intelligence, well-being, sociability). The results showed that our five-dimensional personality summary, which we term the “Predictive Five” (PF), provides predictive performance that is better than the FFM on two independent validation datasets, and on a new set of outcome variables selected by an independent group of psychologists. The approach described herein has the promise of eventually providing an interpretable, low-dimensional personality representation, which is also highly predictive of behavior.

## Introduction

Humans significantly differ from each other. Some people’s idea of fun is partying all night long, and others enjoy binging on a TV series while eating snacks; some are extremely intelligent, and others less so; some are hot-headed, and others remain cool, no matter what. Because of this variety, predicting humans’ thoughts, feelings, and behaviors is a cumbersome task; nonetheless, we attempt to solve this task on a daily basis. For example, when we decide who to marry, we try to predict whether we can depend on the other person till death do us part; when we choose a career, we must do our best to predict whether we will be successful and fulfilled in a given profession.

In order to predict a person’s thoughts, feelings, and behaviors, people often have no other option but to generate something akin to a scientific theory^1^—a parsimonious model that attempts to capture the unique characteristics of individuals, and that could be used to predict their behavior in novel circumstances. Indeed, research shows that people employ such theories when predicting their own^2^ and others’ behaviors. Unfortunately, theories based strictly on intuition are often highly inaccurate^3^, even if produced by professional psychological theoreticians^4^. In light of this, ever since the early days of psychology research, scholars have been attempting to devise personality models using the scientific method, giving rise to the longstanding field of personality science.

Personality, when used as a scientific term, refers to the mental features of individuals that characterize them across different situations, and thus can be used to predict their behavior. In the early years of personality research, scientists generated numerous competing theories and measures, but struggled to arrive at a scientific consensus regarding the core structure of human personality. In recent decades, a consensus theory of the core dimensions of human personality has emerged—the Five Factor Model (FFM).

The FMM emerged from the so-called “lexical paradigm”, which assumed that if people regularly exhibit a form of behavior that is meaningful to human life, then language will produce a term to describe it^5^. Given this assumption, personality psychologists performed research wherein they asked individuals to rate themselves on lists of common English language trait words (e.g., friendly, upbeat), and then developed and used early dimensionality-reduction methods to find a parsimonious model that can account for much of the variability in each person’s trait ratings^5^.

Much research shows that these five factors, often termed the “Big Five” are relatively stable over time and have convergent and discriminant validity across methods and observers^6^. Moreover, research into the FFM has replicated the dimensional structure in different samples, languages, and cultures^7,8^ (but see^9^ for a recent criticism). In light of this, the FFM is taken by some to reflect a comprehensive ontology of the psychological makeup of human beings^10^ according to Mccrae and Costa^11^ the five factors are “both necessary and reasonably sufficient for describing at a global level the major features of personality’’.

Surely, human beings are complex entities, and their personality is not fully captured by five dimensions; however, the importance of having a parsimonious model of humans’ psychological diversity cannot be overstated. As noted by John and Srivasta^12^, a parsimonious taxonomy permits researchers to study *“*specified domains of personality characteristics, rather than examining separately the thousands of particular attributes that make human beings individual and unique.” Moreover, as they note, such a taxonomy greatly facilitate*s “*the accumulation and communication of empirical findings by offering a standard vocabulary, or nomenclature”.

An additional consequence of having a parsimonious model of the core dimensions of human personality, is that such an abstraction enables the acquisition of novel knowledge via statistical learning (see^13^ for a discussion of the importance of abstract representations in learning); namely, whereas the estimation of covariances between high-dimensional vectors is often highly unreliable (i.e., the so-called “curse of dimensionality”^14^), learning the statistical correlates of a low-dimensional structure is a more tractable problem. For example, research has shown that participants’ self-reported ratings on the FFM dimensions can be reliably estimated based on their digital footprint^15^.

This ability to infer individuals’ personality traits using machine learning also raises serious concerns, as it may be used for effective psychological manipulation of the public. In 2013, a private company named *Cambridge Analytica* harvested the data of Facebook users, and used statistical methods to infer the personality characteristics of hundreds of millions of Americans^16^. This psychological profile of the American population was supposedly used by the Trump campaign in an attempt to tailor political advertisements based on an individuals’ specific personality profile. While the success of these methods remains unclear, given the vast amount of data accumulated by companies such as Alphabet and Meta, the potential dangers of machine-learning based psychological profiling is taken by many to be a serious threat to democracy^17^.

Even if dubious entities indeed manage to acquire the Big Five personality profile of entire populations, it is far from obvious that such information could be used to generate actionable predictions. Indeed, the FFM was criticized by some researchers for its somewhat limited contribution to predicting outcomes on meaningful dimensions^18–20^. In light of such claims, some have argued that the public concern over the Cambridge Analytica scandal was overblown^21^ (but see^22^ for evidence for potential reasons for concern).

Roberts et al.^23^ present counter-argument for critical stances against the predictive accuracy of the FFM and note that: “As research on the relative magnitude of effects has documented, personality psychologists should not apologize for correlations between 0.10 and 0.30, given that the effect sizes found in personality psychology are no different than those found in other fields of inquiry.” While this claim is clearly true, there is also no doubt that such correlations (that translate to explained variance in the range of 1%-9%) potentially leave room for improvements in terms of predictive accuracy.

If one’s goal is to find a parsimonious representation of personality that has better predictive accuracy than the FFM, it could be instructive to remember that the statistical method by which the FFM was produced—namely, Factor Analysis—is not geared towards prediction. Factor analysis is an unsupervised dimensionality-reduction method (i.e., a method that maps original data to a new lower dimensional space without utilizing information regarding outcomes) aimed at maximizing explanatory coherence and semantic interpretability, rather than maximizing predictive ability. It does so by finding a parsimonious, low-dimension representation (e.g., the five Big Five factors: extraversion, neuroticism and so on) that maximizes the variance explained in the higher-dimension domain (e.g., hundreds of responses to questionnaire items; for example, “I am lazy”; “I enjoy meeting new people”). Advances in statistics and machine learning have opened up new techniques for *supervised* dimensionality-reduction. Namely, methods that reduce the dimensionality of a source domain (i.e., predictor variables, ${X}_{1},...{,X}_{n}$; in the case of personality, hundreds of questionnaire items) by focusing on the objective of maximizing the capacity of the lower-dimensional representation to *predict* outcomes of a target domain (outcome variables, ${Y}_{1},...{,Y}_{m}$, for example, depression, risky behavior, workplace performance).

Such techniques where dimensionality-reduction is achieved via maximization of predictive accuracy across a host of target-domain outcomes hold the potential of providing psychologists with parsimonious models of a psychological feature space that serve as relatively “generalizable predictors” of important aspects of human behavior. Moreover, it may demonstrate that privacy leaks, a-lá Cambridge-Analytica, are indeed a serious threat to democracy, despite being dismissed by some as science fiction.

In light of this, we investigated whether a supervised dimensionality-reduction approach that takes into account a host of meaningful can potentially improve the predictive performance of personality models. Such an approach could pave the way to a new family of personality models and could advance the study of personality. Alternatively, it may very well be the case that the FFM indeed “carves nature at its joints” and provides the most accurate ontology of the psychological proclivities of humans. In such a case, the FFM may remain the best predictive model of personality, and our approach will not provide improvements in predictions.

In order to examine this question, we conducted three studies. In Study 1, we built a supervised learning model using big data of personality questionnaire items and diverse, important life outcomes. We reduced the dimensionality of 100 questionnaire items into a set of five dimensions, with the objective of simultaneously minimizing prediction errors across ten meaningful life outcomes. We hypothesized that the resulting five-dimensional representation will outperform the FFM representation–when fitting a new model and attempting to predict the ten important outcomes on a held-out dataset. Next, in Studies 2 and 3, we explored the performance of the resulting model on new outcome variables.

## Study 1

### Method

#### Participants

The analyses relied on the myPersonality dataset that was collected between 2007 and 2012 via the myPersonality Facebook application. The myPersonality database is no longer shared by its creators for additional use. We received approval to download that data from the administrators of myPersonality on January 7th, 2018, and downloaded the data shortly thereafter. After the myPersonality database was taken down in 2018, we sent an email to the administrators (on June 8th, 2018), and received confirmation that we can use the data we have already downloaded. The application enabled its users to take various validated psychological and psychometric tests, such as different versions of the International Personality Item Pool (IPIP) questionnaire. Many participants also provided informed consent for researchers to access their Facebook usage details (e.g., liked pages). Participation was voluntary and likely motivated by people’s desire for self-knowledge^24^. The Participants in the myPersonality database are relatively representative of the overall population^25^. All participants provided informed consent for the data they provided to be used in subsequent psychological studies. We used data from 397,851 participants (210,279 females, 142,497 males, and 44,805 did not identify) who answered all of the questions on the 100-item IPIP representation of Goldberg’s^26^ markers for the FFM which are freely available for all types of use. Participants’ mean age was 25.7 years (*SD* = 8.84). The study was approved by the Institutional Review Board of Ben-Gurion University, and was conducted in accordance with relevant guidelines and regulations.

#### Measures

##### Dependent variables

We sought to use supervised learning in order to find a low-dimensional representation of personality that can be used to predict psychological consequences across a diverse set of domains. We thus focused on ten meaningful outcome variables that were available in the myPersonality database, that cover many dimensions of human life which psychologists care about:

(1) Intelligence Quotient (IQ), measured with a brief 20 items version of the Raven’s Standard Progressive Matrices test^27^.

(2) Well-being, measured with the Satisfaction with Life scale^28^.

Personal values, measured using two scores representing the two axes from the Schwartz's Values Survey:

(3) Self-transcendence vs. Self-enhancement values and

(4) Openness to Change vs. Conservation values^29^.

(5) Empathy, measured with the Empathy Quotient Scale^30^.

(6) Depression, measured with The Center for Epidemiologic Study Depression (CES-D) scale^31^.

(7) Risky behavior, measured with a single-item question concerning illegal drug use.

(8) Self-reports of legal, yet unhealthy behavior (measured as averaging two single-item questions concerning alcohol consumption and smoking).

(9) Single item self-report of political ideology.

(10) The number of friends of participants’ had on the social network Facebook.

##### Independent variables

Our independent variables were the participants’ answers to the 100 questions included in the IPIP-100 questionnaire^32^. In this questionnaire, the participants are asked to rate their agreement with various statements related to different behaviors in their life and their general characteristics and competencies, on a scale from 1 (strongly disagree) to 5 (strongly agree). The original use of this questionnaire is to reliably gauge participants' scores on each of the FFM dimensions. It includes five subscales, each containing 20 items; the factor score for each FFM dimension can be calculated as a simple average of these 20 questions (after reverse coding some items). In the current research we treat each item from this list of 100 questions as a separate independent variable, and seek to reduce the dimensionality of this vector using supervised learning.

#### Model construction

The problem we set out to solve is to find a good predictive model that is: (a) based on the 100 questions of the existing IPIP-100 questionnaire, and (b) uses five variables only, so we can fairly compare it with the FFM. Reduced Rank Regression (RRR) is a tool that allows just that: it can be used to compress the original 100 IPIP items, to a set of five new variables. These new variables are constructed so that they are good predictors, on average, of a large set of outcomes. Unlike Principal Component Analysis (PCA) or Factor Analysis, RRR reduces data dimensionality by optimizing predictive accuracy.

We randomly divided our data into an independent train and test sets. Each subject in the train and test set had 100 scores of the IPIP questionnaire (${X}_{1},{X}_{2},...{,X}_{100}$), as well as their score in each of the ten dependent variables (${Y}_{1},{Y}_{2},...{,Y}_{10}$).

*X* (*n* × 100) and *Y* (*n* × 100) have been centered and scaled. We fitted a linear predictor, with coefficient vector:

1 $${\widehat{Y}}_{j} := \sum_{k=1}^{100} {X}_{k}{C}_{kj}\quad j= {1,2},....10 $$

And in matrix notation:

2 $$\widehat{Y} = XC $$

Our linear predictors were fully characterized by the matrix C. We wanted these predictors to satisfy the following criteria: (a) minimize the squared prediction loss (b) consist of 5 predictors, i.e., *rank*(*C*) = *r* = 5. Criterion (a) ensures the goodness of fit of the model, and criterion (b) ensures a fair comparison with the FFM. The RRR problem amounts to finding a set of predictors, $\hat{C}$, so that:

3 $$ \hat{C}: = argmin_{C} \left\{ { \left| {\left| { Y - XC } \right|} \right| ^{2 } , such\; that \;rank\left( C \right) = r} \right\}, $$

where || $\cdot$ || denotes the Frobenius matrix norm. The matrix $C$ can be expressed as a product of two rank-constrained matrices:

4 $$C := B{A}^{T}$$

where $B$ is of has p rows and r columns, denoted, *p* × *r*, and ${A}$ is of dimension *q* × *r*. The model (2) may thus be rewritten as:

5 $$\widehat{Y} = (X\widehat{B}){\widehat{A}}^{T}{ }$$

The *n* × *r* matrix $X\hat{B}$, which we noted $\tilde{X}$, may be interpreted as our new low-dimension personality representation. Crucially for our purposes, the same set of *r* predictors is used for all dependent variables. By choosing dependent variables from different domains, we dare argue that this set of predictors can serve as a set of “generalizable predictors”, which we call henceforth the *Predictive Five* (PF). For the details of the estimation of $\hat{B}$ see the attached code. For a good description of the RRR algorithm see^33^.

#### Model assessment

To assess the predictive performance of the PF, and compare it to the predictive properties of the classical FFM, we used a fourfold cross validation scheme. The validation worked as follows: we learned $\hat{B}$ from a train set (397,851 participants) using RRR; we then divided the independent test set (800 participants) into 4 subsets; we learned $\hat{A}$ from a three-quarters part of the test set (600 participants), and computed the *R*^2^ on the holdout test set (200 participants); we iterated this process over the 4-test subsets. The rationale of this scheme is that: (a) predictive performance is assessed using *R*^2^ on a completely *novel dataset*; (b) when learning the predictive model, we wanted to treat the personality attributes as known. We thus learned $\hat{B}$ and $\hat{A}$ from different sets. The size of the holdout set was selected so that *R*^2^ estimates will have low variance. The details of the process can be found in the accompanying code (https://github.com/GalBenY/Predictive-Five).

To examine the performance of the RRR algorithm against another candidate reference model we also performed Principal Component Regression (PCR), where we reduced the IPIP questionnaire to its 5 leading principal components, which were then used to predict the outcome variables. We used the resulting model as a point of comparison in follow-up assessment of predictive accuracy. Like the RRR case, we learned the principal components from the train-set (397,851 participants). Next we divided the independent test set (800 participants) into 4 subsets and used a fourfold cross validation: ¾ to learn 5 coefficients, and ¼ to compute.

In order to calculate the significance of the difference in the predictive accuracy of the models we took the following approach: predictions are essentially paired, since they originate from the same participant. For each participant, we thus computed the (holdout) difference between the (absolute) error of the PF and FFM models: $|{{\widehat{y}}_{i}}^{PF}|-|{{\widehat{y}}_{i}}^{FFM}|$. Given a sample of such differences, comparing the models collapses to a univariate t-test allowing us to reject the null hypothesis that the mean of the differences is 0.

### Results

#### PF loadings

**Figure 1:**
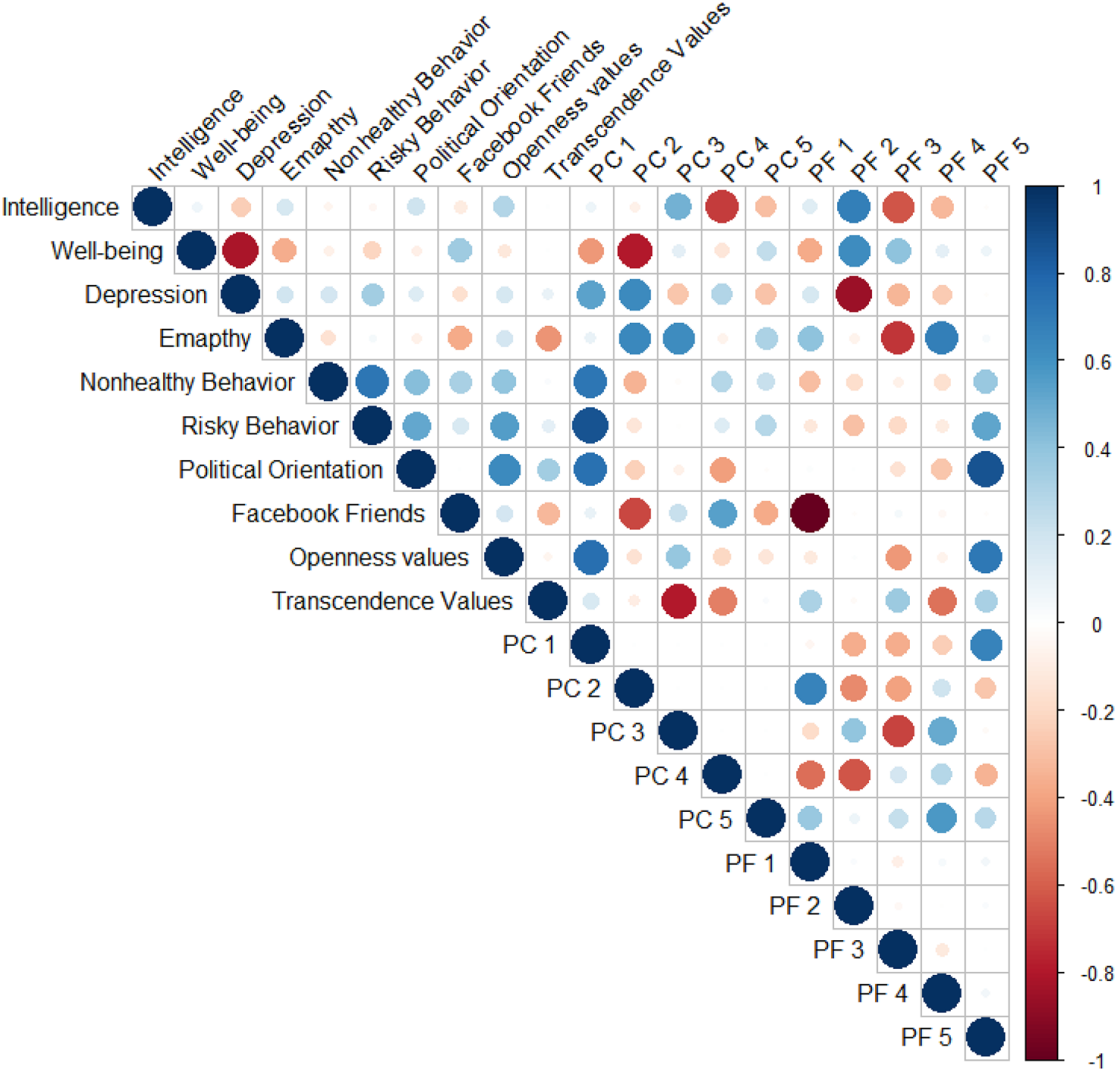
Correlations between the 10 outcome variables, 5 principle components of outcome variables, and the 5 PF dimensions.

**Figure 2:**
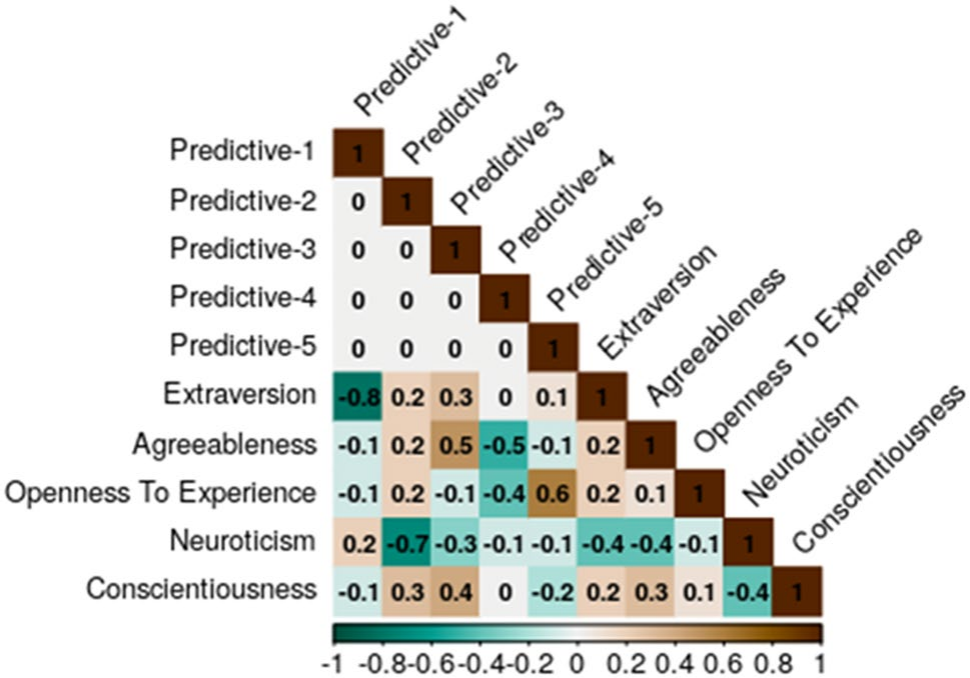
Correlations between the PF and FFM scale scores.

Each of the resulting PF dimensions were a weighted linear combination of IPIP-100 item responses. Despite the fact that the resulting model was based on a questionnaire meant to reliably gauge the FFM, the resulting outcome did *not* fully recapitulate the FFM structure. The detailed loadings for each of the resulting five dimensions appears in the supplementary materials (Fig. 1, Supplementary Materials), can be examined in an online application we have created (https://predictivefive.shinyapps.io/PredictiveFive), and can be easily gleaned by examining the correlation of PF scores to the FFM scores (Fig. 2). None of the PF dimensions strongly correlated with demographic variables (Table 1, Supplementary Materials). In Fig. 1, we display the correlations between the ten outcome variables, five principle components of these outcome variables (capturing 86% of the total variance), and the five PF dimensions. For example, it can be observed the PF 3 is inversely related to performance on the intelligence test and to empathy.

**Table 1:**
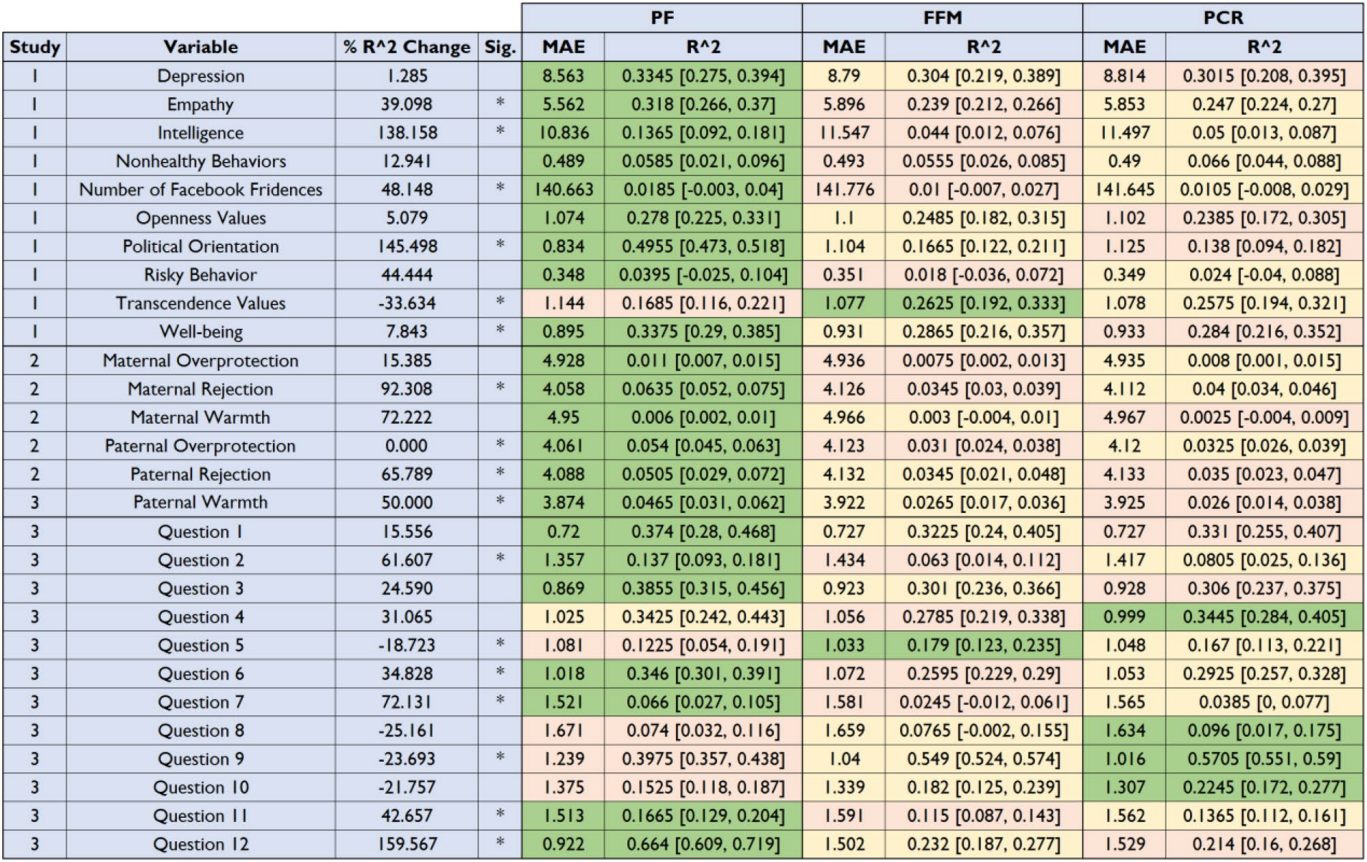
Comparison of the predictive performance of the different models.

PF—Performance of the model based on the Predictive Five representation; FFM—performance of model based on the Five Factor Model representation; PCR—performance of the Principal Component Regression model; MAE—Mean Absolute Error. For each outcome variable, the most successful model (in terms of predictive accuracy) is colored in green, the second-best model is colored in orange, and the least successful model is colored in pink. Percent *R*^2^ change and significance pertains to the focal comparison (PF-based MAE vs. FFM-based MAE). Significant focal comparisons are denoted by an asterisk, and survive a threshold of *p* < .05, FDR corrected. In 14 cases, the PF-based model significantly outperformed the FFM-based model, in 3 cases, the FFM-based model was significantly better than the PF-based model.

#### Predictive performance

The out-of-sample *R*^2^ of the three models is reported in Table 1. From this figure, we learn that the PF-based regression model is a better predictor of the outcome variables. This holds true on average (over behavioral outcomes), but also for nine of the ten outcomes individually. On 5 of the 10 comparisons, the PF-based model significantly outperformed the FFM, and in a single case the FFM-based model significantly outperformed the PF. The average improvement across all 10 measures was 40.8%.

#### Reproducibility analysis

If it were the case that our model discovery process produces very different loadings when run on different samples of participants, then the ontological status of the PF representation should be called into question.

In order to assess the reproducibility of the PF we split the training dataset from Study 1 into two datasets; sample A with 198,850 participants and sample B with 198,851 participants. We then learned the rotation matrix, B, on each data part, and applied it. Equipped with two independent copies of the PF, ${X}_{l }{\widehat{B}}_{l}, l=\{A,B\}$ replicability is measured by the correlation between data-parts, over participants. Table 2 reports this correlation, averaged over the 5 PFs (column “Correlation between replications”). As can be seen, the correlation between the replications is satisfactory-to-high and ranges from 0.7 to 0.98. This suggests that PF representation replicates well across samples.

**Table 2:** Reproducibility and reliability analysis.

	Test–retest correlation	Correlation between replications
Dimension 1	0.79	0.98
Dimension 2	0.75	0.77
Dimension 3	0.69	0.70
Dimension 4	0.71	0.79
Dimension 5	0.79	0.86

Test–retest correlation represents the test–retest reliability within participants. The correlation between replications captures the reproducibility of our dimensional structure across participants.

#### Reliability analysis

If the same individuals, tested on different occasions, receive markedly different scores on the PF dimensions, then the ontological status of the PF representation should be called into question. To this end, we exploit the fact that 96,682 users answered the IPIP questionnaire twice. The test–retest correlation between these two answers is reported in Table 2 (column “Test–retest correlation”). It varied from 0.69 for the Dimension 3, to 0.79 for both Dimensions 1 and 5, suggesting that the variance captured by these dimensions is indeed (relatively) stable.

#### Divergence from the FFM

The superior predictive performance of the PF representation provides evidence that it differs from the FFM. Additionally, as can be gleaned from Fig. 2 (and from the detailed factor loadings’ Supplemental Material), Dimensions 3 and 4 reflect a relatively even combination of several FFM dimensions.

However, these observations do not provide us with an estimate of the degree of agreement between the two *multidimensional* spaces. Prevalent statistical methods of assessment of discriminant validity^34^ are also not suitable to answer our question regarding the convergence\divergence between the PF and FFM spaces. These various methods only provide researchers with estimates of the agreement between *unidimensional constructs*.

Nonetheless, the underlying logic behind these methods (i.e., a formalization of a multitrait-multimethod matrix^35^) is still applicable to our case. We calculated an estimate of agreement between the FFM and the PF spaces using *cosine similarity*, which gauges the angle between two points in a multidimensional space (the smaller the angle, the closer are the points). Our rationale is that if the FFM scores differ from the PF, they should span different spaces. The cosine similarity *within* measures (in our case, first and second measurements, denoted T1 and T2) should thus be larger than the similarity *between* measures (FFM to PF).

We used the data from the 96,682 participants for which we had test–retest data. Instead of computing standard test–retest correlations, we calculated a multidimensional test-rest score as the cosine similarity of participants’ scores on the first and second measurement, for both the FFM and PF. These estimates are expected to be highly similar and provide an upper bound on the similarity measure, partially analogous to the diameter of the multitrait-multimethod matrix. In a second stage, for each T1 and T2 vector, we measured the extent to which participants’ FFM scores are similar to their PF score, thereby calculating a magnitude that is analogous to measures of *divergent validity*. Because cosine similarity is sensitive to the sign and order of dimensions, we extracted the maximal possible similarity between the two spaces, providing the most conservative estimate of divergent validity.

**Figure 3:**
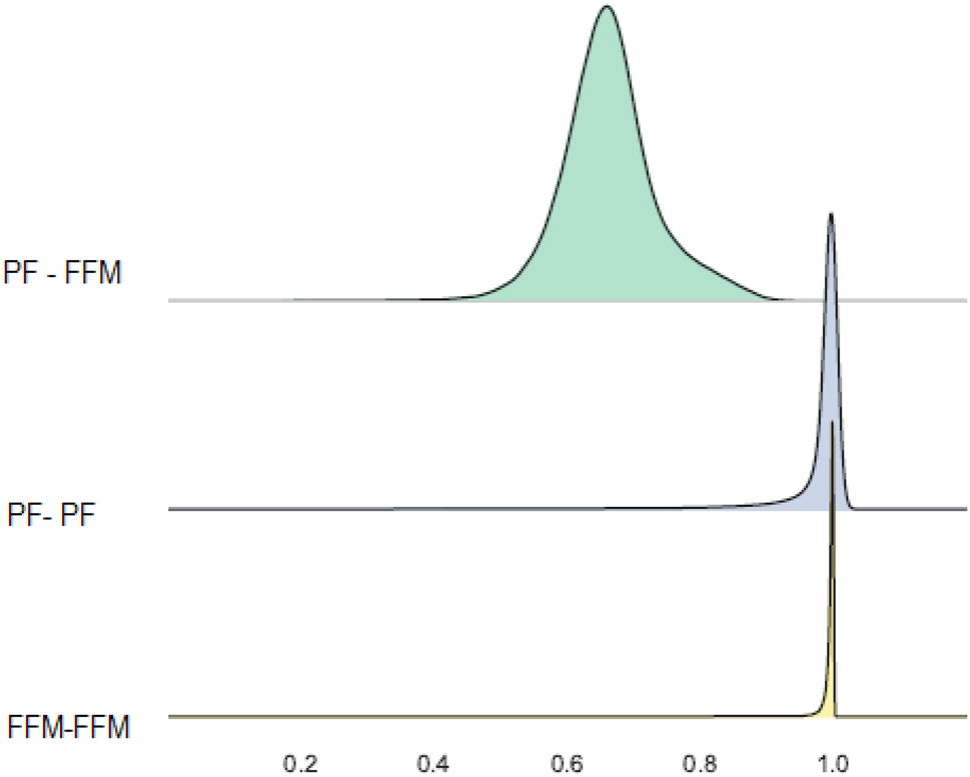
Distribution, over participants, of the multidimensional similarity between the FFM and PF representations.

As can be seen in Fig. 3, the T1-T2 similarity of the FFM is nearly maximal (*M* = 0.994, *SD* = 0.011); the T1-T2 similarity of PF is also very high (*M* = 0.969, *SD* = 0.100). The similarity between the FFM and the PF on both T1 and T2 is much lower (*M* = 0.730, *SD* = 0.111). The minimal difference between the convergence measures and divergence measures is on the magnitude of Hedge's *g* of 2.217, clearly representing a substantial divergence between the FFM and PF spaces. In other words, while the PF representation bears some resemblance, it is clearly a different representation.

### Discussion

The results of Study 1 provide evidence that a supervised dimensionality reduction method can yield a low-dimensional representation that is simultaneously predictive of a set of psychological outcome variables. We demonstrate that by using a standard personality questionnaire and supervised learning methods, it is possible to improve the overall prediction of a set of 10 important psychological outcomes, even when restricting ourselves to 5 dimensions of personality. RRR allowed us to compress the 100 questions of the personality questionnaire to a new quintet of attributes that optimize prediction across a large set of psychological outcomes. The resulting set of five dimensions differs from the FFM, and has better predictive power on the held-out sample than the classical FFM and an additional comparison benchmark of five dimensions generated using Principal Component Analysis.

A theory of personality should strive to predict humans’ thoughts, feelings, and behaviors across different life contexts. Indeed, the representation we discovered in Study 1 was superior to the FFM in terms of its ability to predict a diverse set of psychological outcomes on a set of novel observations. The fact that the same low-dimensional representation was applicable across a set of important outcomes of human psychology suggests that it is a relatively generalizable model, in the sense that it simultaneously applies to several important domains. However, despite the diversity of the outcome measures examined in Study 1, it remains possible that the PF representation is only effective for the prediction of the set of outcome measures on which it was trained. Such a finding would not negate the usefulness of this model, given the wide variety of outcomes captured by the PF. However, it is interesting to see whether the resulting representation can improve prediction on additional sets of outcomes. In light of this, in Study 2 we sought to examine the performance of the PF on a set of novel outcome measures that were present in the myPersonality database, but that were held-out from the model generation process. Specifically, in this study we sought to see whether the PF representation outperforms the FFM in its ability to predict participants’ experiences during their *childhood*.

Unlike the outcome measures used in Study 1, this dependent variable does not pertain to participants’ lives in the present, rather, it is a measure of their past experiences. As such, “retrodiction” of remote history may be especially challenging. Nonetheless, it is widely held that individuals’ psychological properties are shaped, at least to some extent, by the degree to which they were raised in a loving household^36,37^. Indeed, there is evidence to the fact that many specific psychological attributes are shaped by experiences with primary caregivers (e.g., shared environmental effects on topics such as food preference^38^, substance abuse^39^, and agression^40^). In light of this, we reasoned that it is reasonable to expect that one's personality profile should contain information that is predictive of individuals' retrospective reports of their upbringing.

## Study 2

### Method

#### Participants

We used data from 3869 participants who answered all of the questions on the 100-item IPIP representation of^26^ markers for the Big Five factor structure, and answered the short form “My Memories of Upbringing” (EMBU) questionnaire^41^.

#### Measures

The short form of the EMBU includes a total of six subscales: three subscales that contain questions to measure the extent to which the participants' *father* was a *warm*, *rejecting*, and *overprotecting* parent, and three subscales that measure the extent to which the participants' *mother* was *warm*, *rejecting*, and *overprotecting*.

### Results

As can be seen in Table 1, for all six variables, prediction accuracy was relatively low; however, importantly, in all six cases the PF-based model outperformed the FFM-based model, and was significantly better for four out of the six outcome variables. The average improvement across the six outcome measures was 49.2%.

### Discussion

The results of Study 2 further support the idea that the PF representation that was built using the 10 meaningful outcome measures present in the myPersonality database is at least somewhat generalizable. However, Study 2 again relied on myPersonality participants, upon which the PF was built. In light of this, in Study 3 we sought to further test the generality of the PF by examining whether it outperforms the FFM-based model on a set of new participants. Furthermore, we wanted to see whether our model can outperform the FFM-based model on a set of new outcome measures selected by an independent group of professional psychologists, blind to our model-generation procedure.

## Study 3

### Method

#### Participants

We collected new data using Amazon’s Mechanical Turk (www.MTurk.com). M-Turk is an online marketplace that enables data collection from a diverse workforce who are paid upon successful completion of each task. Our target sample size was 500 participants, which is double the size of what is considered a standard, adequate sample size in individual differences research^42^. In practice, 582 participants participated in the study, 35 of them were omitted for failing attention checks, leaving 547 participants in the final dataset (243 females and 304 men). This number exceed a sample size of 470 participants which provides 95% confidence that a small effect (⍴ = 0.1) will be estimated with narrow (w = 0.1) Corridor of Stability^42^.

#### Measures

##### Dependent variables

In order to make sure that the PF generalize across different domains of psychological interest, it was important to generate the list of outcome variables in a way that is not biased by our knowledge of the original ten outcome variables on which the PF was designed (i.e., intelligence, well-being, and so on). Therefore, on January 3rd, 2019, we gathered a list of 12 new outcome measures by posting a call on the Facebook group PsychMAP (https://www.facebook.com/groups/psychmap) asking researchers: “to name psychological outcome measures that you find interesting, important, and that can be measured on M-Turk using a single questionnaire item on a Likert scale.” Once we arrived at the target number of questions we closed the discussion and stopped collecting additional variables. The 12 items were suggested by eight different psychologists, six of which had a PhD in psychology and five were principal investigators. By using this variable elicitation method, we had no control over the outcome measures, and could be certain that we have gathered a randomly-chosen sample of outcomes that are of interest to psychologists.

This arbitrariness of the outcome generation process (selecting the first 12 outcomes nominated by psychologists, without any consideration of consensus views regarding variable importance)—and the likely low psychometric reliability of single-item measures–can be seen as a limitation of this study. However, our reasoning was that such a situation best approximates the "messiness" of the unexpected, noisy, real-world scenarios wherein prediction may be of interest–and as such, provides a good test of predictive performance of the FFM and PF.

In the M-turk study, participants rated their agreement with 12 statements (1- Strongly Disagree to 7- Strongly Agree). The elicited items were:

(1) “I care deeply about being a good person at heart”.

(2) “I value following my heart/intuition over carefully reasoning about problems in my life”.

(3) “Other people's pain is very real to me”.

(4) “It is important to me to have power over other people”.

(5) “I have always been an honest person”.

(6) “When someone reveals that s/he is lonely I want to keep my distance from him/her”.

(7) “Before an important decision, I ask myself what my parents would think”.

(8) “I have math anxiety”.

(9) “I am typically very anxious”.

(10) “I enjoy playing with fire”.

(11) “I am a hardcore sports fan”.

(12) “Politically speaking, I consider myself to be very conservative”.

##### Independent variables

The independent variables were participants’ answers to the 100 questions of the IPIP questionnaire.

#### Model assessment

Similarly to Study 1, we use a fourfold cross validation scheme in order to assess the predictive performance of the PF on new data set and outcome variables. Next, we compared it to the predictive performance of the FFM. The validation worked as follows: we had $\hat{B}$ from Study 1, we learned $\hat{A}$ from a part of the new sample (400 ~ participants) and computed the *R*^2^ on the holdout test set (130 ~ participants). In the spirit of the fourfold cross-validation, we iterated this process over the 4-test sets and calculated the average test *R*^2^ for each model.

### Results

Similarly to Studies 1–2, the results showed that the predictive performance of the PF was again better than that of the Big Five, although the improvements were more modest (average 30% improvement across the 12 measures). In 5 out of 12 cases, the PF-based model was significantly better than the FFM-based model, and the opposite was true in 2 cases.

### Discussion

The out-of-sample *R*^2^ of the two models (PF\Big Five) in Study 3 show a consistent trend with the results presented earlier in Study 1 and Study 2, that is, a somewhat higher percentage of explained variance in the models with the PF as predictors. This improvement observed in Study 3 was more modest than that observed earlier, but is nonetheless non-trivial—given that the set of outcome variables was different from the one the PF representation was trained on, and given that the PF representation was trained on items from questionnaires designed to measure the FFM. As such, the results of Studies 1–3 clearly demonstrate the generalizability of the PF.

A potential criticism of these findings is that the success of the PF model was more prominent on variables that were more similar to the 10 dependent measures upon which the PF was trained. However, it is important to keep in mind that the 12 outcome measures in this study were selected at random by an external group of psychologists. As such, this primarily means that the 10 psychological outcomes used to train the PF indeed provide good coverage of psychological processes that are of interest to psychologists, and thereby, overall, generalize well to novel prediction challenges.

## General discussion

In this contribution, we set out to examine the viability of a novel approach to modeling human personality. Unlike the prevailing Five-Factor Model (FFM) of personality, which was developed by relying on *unsupervised* dimensionality reduction techniques (i.e., Factor Analysis), we utilized *supervised* machine learning techniques for dimensionality reduction, using numerous psychologically meaningful outcomes as data labels (e.g., intelligence, well-being, sociability). Whereas the FFM is optimized towards discovering an ontology that explains most of the variance on self-report measures of psychological traits, our new approach devised a low-dimensional representation of human trait statements that is optimized towards *prediction* of life outcomes. Indeed, the results showed that our model, which we term the Predictive Five (PF), provides predictive performance that is better than the one achieved by the FFM in independent validation datasets (Study 1–2), and on a new set of outcome variables, selected independently of the first study (Study 3). The main contribution of the current work is explicating and demonstrating a methodological approach of generating a personality representation. However, the results of this work is a specific representation that is of interest and of potential use in and of itself. We now turn to discuss both our general approach and the resulting representation.

### Interpreting the PF

The dimensional structure that emerged when using our supervised-dimensionality reduction approach differed from the FFM. Two dimensions (Dimension 1 and 2) largely reproduced the original FFM factors of Extraversion and Neuroticism. Interestingly, these two dimensions are the ones that were highlighted in early psychological research as the “Big Two” factors of personality (Wiggins, 1966). Dimension 5 was also highly related to an existing FFM dimension, namely, *Openness to Experience*.

The third and fourth dimensions in the model did not correspond to a single FFM trait, but were composed of a mixture of various items. An inspection of the loadings suggests that Dimension 4 is related to some sort of a combative attitude, perhaps captured best by the construct of Dominance^44–45^. The items that loaded highly on this dimension related to hostility (“Do not sympathize with others”; “Insult people”), a right-wing political orientation (“Do not vote for liberal political candidates”), and an approach-oriented^46^ stance (“Get chores done right away”; “Find it easy to get down to work”).

Like PF Dimension 4, Dimension 3 also seemed to capture approach-oriented characteristics (with high loadings for the items “Get chores done right away” and “Find it easy to get down to work”), however, this dimension differed from Dimension 4 in that it represented a harmony-seeking phenotype^47^. The items highly loaded on this dimension were those associated with low levels of narcissism (“keep in the background”, “do not believe I am better than others”) but with a stable self-worth (“am pleased with myself”). Additional items that were highly loaded on this dimension were those that reflect cooperativity (“concerned with others” and “sympathize with others”).

These two dimensions may seem like dialectical opposites. Indeed, the item “sympathize with others” strongly loaded on both factors, but with a different sign. However, the additional items that strongly loaded on these two dimensions appear to have provided a context that altered the meaning of this item. This is evident in the fact that Dimensions 3 and 4 are not correlated with each other. A possible speculative interpretation is that the two phenotypes captured by Dimensions 3 and 4 can be thought of as two strategies that may have been adaptive throughout human evolution. The first, captured by Dimension 4 seems to represent aggressive traits that may have been especially useful in the context of *inter*-group competition and conflict; the second, captured by Dimension 3, seems to represent traits that may be associated with *intra*-group cooperation and peace.

In general, the interpretability of the PF representation is lower than that of the FFM, with some surprising items loaded together on the same dimension. For example, the two agreeableness items that “do not believe I am better than others” and “respect others” that are strongly correlated with each other were highly loaded onto Dimension 1 (that is related to introversion), but with opposite signs. To a certain extent, this is a limitation of the predictive approach in psychology. However, such confusing associations may lead us towards identifying novel insights. For example, it is possible that some individuals adopt an irreverent stance towards both self and others, and such a stance could be predictive of various psychological outcomes, and correlated with introversion.

### Towards a more predictive science of personality

As noted, the reasons that people seek models of personality are twofold: first, we want models that allow us to understand, discuss and study the differences between people; second, we need these models in order to be able to predict and affect people’s choices, feelings and behaviors^48^. Current approaches to personality modeling succeeded on the former, providing highly comprehensible dimensions of individual differences (e.g., we can easily understand and communicate the contents of the dimension of “Neuroticism” by using this sparse semantic label). However, the ability of the FFM to accurately predict outcomes in people’s lives is at least somewhat limited^19–20,49^.

The significance of the current work is that it describes a new approach to modeling human personality, that makes the prediction of behavior an explicit and fundamental goal. Our research shows that supervised dimensionality reduction methods can generate relatively generalizable, low-dimensional models of personality with somewhat improved predictive accuracy. Such an approach could complement the unsupervised dimensionality reduction models that have prevailed for decades in personality research. Moreover, this research can complement attempts to improve the predictive validity of psychology by using non-parsimonious (i.e., facets and item-level) questionnaire-based predictive models^50^.

Aside from providing a general approach for the generation of personality models, the current research also provides a potentially useful instrument for psychologists across different domains of psychological investigation. Our findings suggest that psychologists who are interested in predicting meaningful consequences (e.g., workplace or romantic compatibility) or in optimizing interventions on the basis of individuals’ characteristics (e.g., finding out which individuals will best respond to a given therapeutic technique)—may benefit from incorporating the PF dimensions in their predictive models. To facilitate such future research, we provide the R code that calculates the five dimensions based on answers on the freely available IPIP-100 questionnaire (https://github.com/GalBenY/Predictive-Five). The use of an existing, open-access, widely-used questionnaire means that researchers can now easily apply the PF coding scheme alongside with the FFM coding scheme to their data, and compare the utility of the two models in their own specific research domains.

One avenue of potential use of the PF representation is in clinical research. The PF showed improved prediction of depression and well-being; moreover, the PF substantially outperformed the FFM in the prediction of two known resilience factors (intelligence and empathy). Specifically, PF Dimension 3 (which, as noted above, seems to represent some harmony-seeking phenotype) significantly contributed to the prediction of all of four outcomes. As such, future work could further investigate the incremental validity of this dimension (and the PF representation more generally) as a global resilience indicator.

Across a set of 28 comparisons, the predictions derived from the PF-based model were significantly better in 15 cases, and significantly worse in 3 cases. The average improvement in *R*^2^ across the 28 outcomes was 37.7%. However, it is important to note that the PF representation described herein is just a first proof of concept of this general approach, and it is likely that future attempts that are untethered to the constraints undertaken in the current study can provide models of greater predictive accuracy. Specifically, in the current research we relied on the IPIP-100, a questionnaire designed by researchers specifically in order to reliability measure the factors of the FFM, and limited ourselves to a five-dimension solution, to allow comparison with the FFMs. The PF representation outperformed the FFM representation despite these constraints. These results provide a very conservative test for the utility of our approach.

### Future directions

Future attempts to generate generalizable predictive models will likely produce even stronger predictive performance if they relax the constraint of finding exactly five dimensions and perform dimensionality-reduction based on the raw data used to generate the FFM itself—namely, the long list of trait adjectives that exist in human language, and that were reduced into the five dimensions of the FFM.

For the sake of simplicity comparability to the FFM, the current work employed a linear method for supervised dimensionality reduction. Recent work in machine learning has demonstrated the power of Deep Neural Networks as tools for dimensionality reduction (e.g., language embedding models). In light of this, it is likely that future work that utilizes non-linear methods for supervised dimensionality reduction could generate ever more predictive representations (i.e., “personality embeddings”).

A limitation of the current work is that the PF was trained on a relatively limited set of 10 important life outcomes (e.g., IQ, well-being, etc.). While these outcome measures seem to cover many of the important consequences humans care about (as evident by the predictive performance on Study 3), it is likely that training a PF model on a larger set of outcome variables will improve the coverage and generalizability of future (supervised) personality models. A potential downside of extending the set of outcome measures used for training, is that at some point (e.g., 20, 100 outcomes) it is possible that the “blanket will become too short”: namely, that it will be difficult to find a low-dimensional representation that arrives at satisfactory prediction performance simultaneously across all outcomes. Thus, future research aiming at generating more predictive personality models may need to find a “sweet spot” that allows the model to fit to a sufficiently comprehensive array of target outcomes.

What may be the most important consequence of the current approach is that whereas previous attempts of modeling human personality necessarily limited by their reliance on the subjective products of the human mind (i.e., were predicated on human-made psychological theories, or subjective ratings of trait words), our approach holds the unique potential of generating personality representations that are based on objective inputs.

A final question concerning predictive models of personality is whether we even want to generate such models, given the potential of their misuse. While the current results still show the majority of variance in psychological outcomes remain unexplained–in the era of social networks and commercial genetic testing, the predictive approach to personality modeling could theoretically lead to models that render human behavior highly predictable. Such models give rise to both ethical concerns (e.g., unethical use by governments and private companies, as in the Cambridge-Analytica scandal) and moral qualms (e.g., if behavior becomes highly predictable, what will it mean for notions of free will and personal responsibility?). While these are all valid concerns, we believe that like all other scientific advancements, personality models are tools that can provide a meaningful contribution to human life (e.g., predicting suicide in order to avoid it; predicting which occupation will make a person happiest). As such, the important, inescapable quest towards generating even more effective models that will allow us to predict and intervene in human behavior is only just the beginning.

## Supplementary Information

Supplementary Information.

## Data Availability

The data for Study 1, 3 and 4 rely on the myPersonality database (www.mypersonality.org) which is an unprecedented big-data repository for psychological research, used in more than a hundred publications. We achieved permission from the owners of the data to use it for the current research—but we do not have their permission to share it for wider use. The data for Study 2 is available upon request. We also share the complete code and the full model with factor loadings (https://github.com/GalBenY/Predictive-Five).
