# Supplementary material for: A prediction-focused approach to personality modeling
Source: Sci Rep. 2022 Jul 25;12:12650. doi: 10.1038/s41598-022-16108-3 (PMC9314364; doi:10.1038/s41598-022-16108-3)

Supplementary Materials

Demographic analysis

In order to better understand the relation between the PF dimensions and demographic variables, we analyzed the correlations between these five dimensions and age, ethnicity, and gender.

Age. The analysis of correlations between age and FFM\PF scores relied on 122,010 myPersonality participants for which we had age data.

Gender. The gender analysis relied on 352,776 participants from the myPersonality database for which we had information regarding gender identification (210,279 males, 142,497 females). Participants' gender was analyzed as an independent dummy variable, coded as 0 for male and 1 for female, FFM and PF scores were treated as the dependent variable.

Ethnicity. The analysis concerning ethnicity was based on the sample from Study 3 wherein we had accurate ethnicity information. The data included 547 participants; 102 participants who identified as black (and dummy coded as 0) and 445 participants who identified as white (dummy coded as 1). Again, like in the gender analysis, ethnicity was treated as the independent variable, and PF and FFM scores were the dependent variable.

As can be seen in Table 1, for *Age*, the maximal R^2 for PF dimensions was 0.085 (for PF 4), and the maximal R^2 for the FFM was 0.157 for (Conscientiousness). For *Gender*, the maximal PF R^2 was 0.04 (PF 2), and the maximal FFM R^2 was 0.032 (Neuroticism). For *Ethnicity*, the maximal PF R^2 was 0.014 (PF 5), and the maximal FFM R^2 was 0.016 (Agreeableness).

Table 1. R^2 of linear models predicting age, gender, and ethnicity by FFM and PF dimensions.

	Age	Gender	Ethnicity
**PF Dimension 1**	0.001	0.002	0.003
**PF Dimension 2**	0.006	0.033	0.004
**PF Dimension 3**	0.007	0.04	0.001
**PF Dimension 4**	0.007	0.01	0.013
**PF Dimension 5**	0.00	0.0005	0.014
**Openness**	0.002	0	0
**Conscientiousness**	0.024	0.0024	0.004
**Extraversion**	0	0.002	0.0001
**Agreeableness**	0.005	0.0131	0.0164
**Neuroticism**	0.002	0.032	0.0001

PF Dimension Loadings

Figure 1a. PF Dimension 1

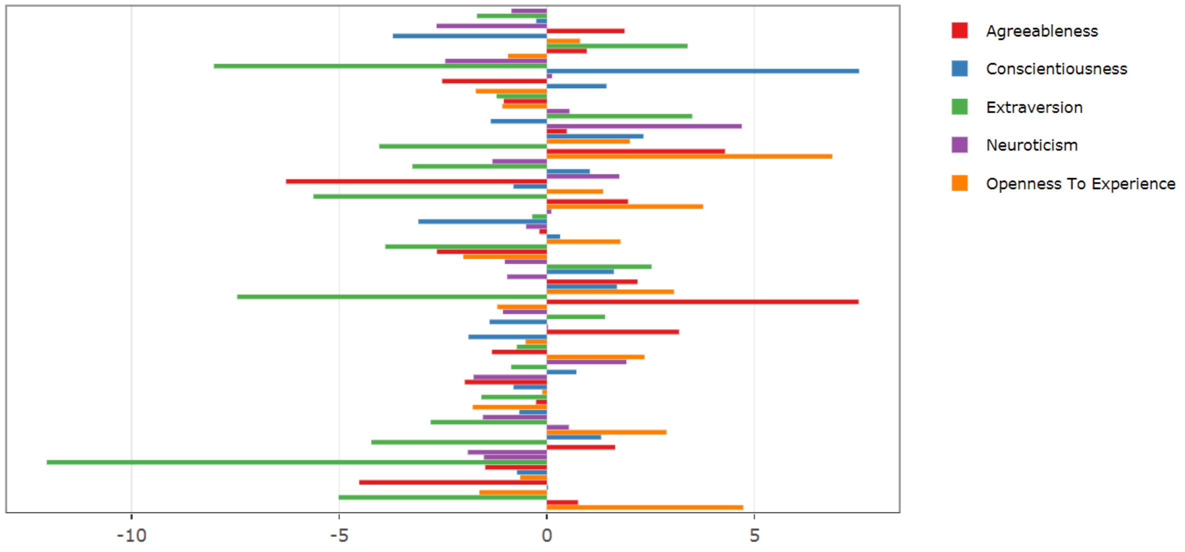

Figure 1b. PF Dimension 2

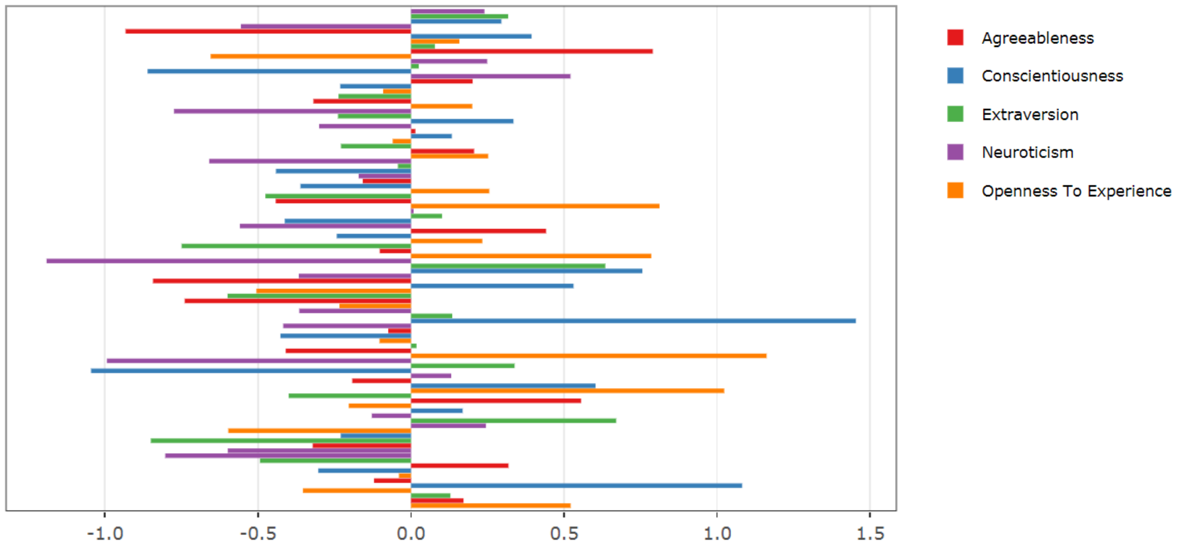

Figure 1c. PF Dimension 3.

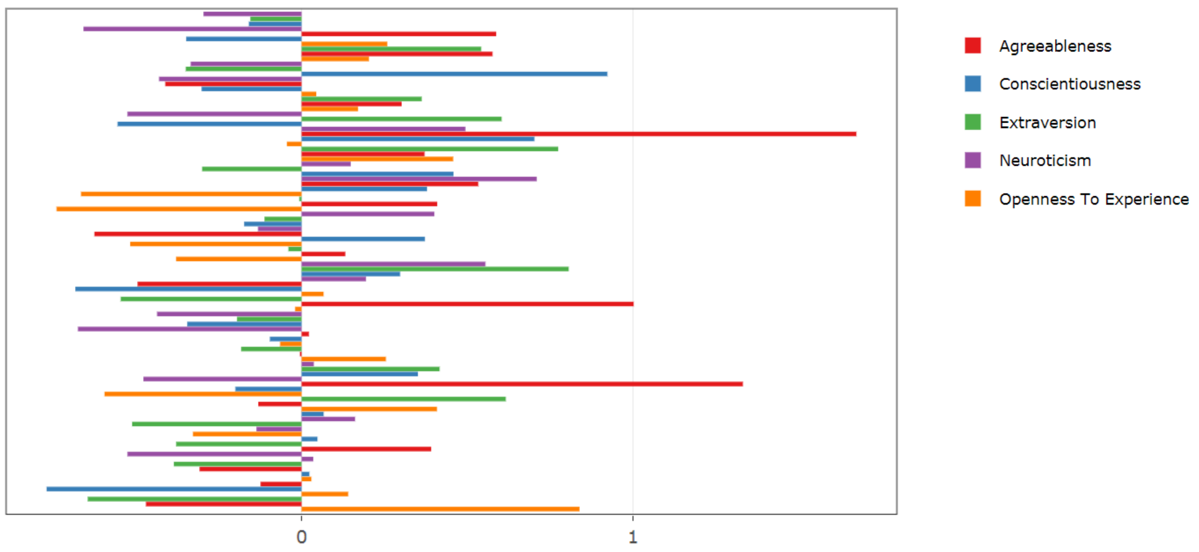

Figure 1d. PF Dimension 4

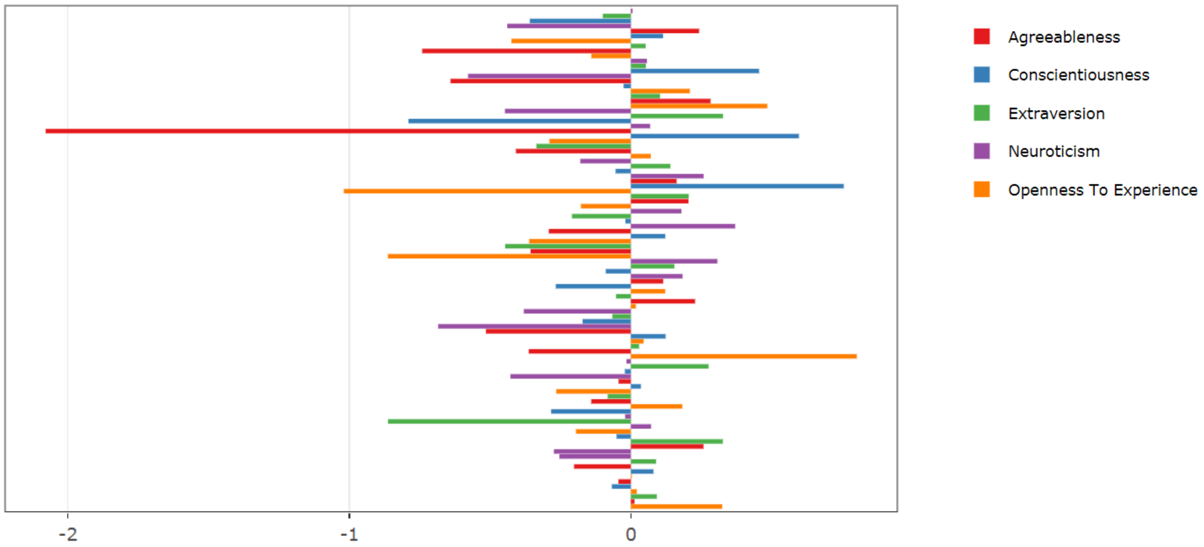

Figure 1e. PF Dimension 5.

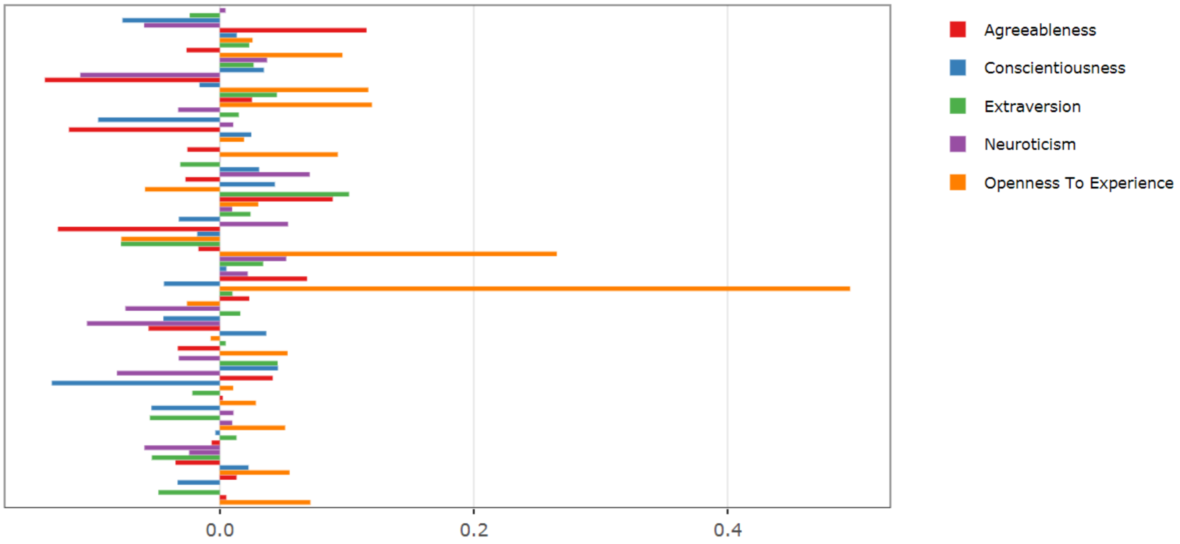

Supplement: Supplementary file 1 — Supplementary Information. [file 41598_2022_16108_MOESM1_ESM.pdf]
